# A Single-step Generation of AlissAID-based Conditional Knockdown Strains Using Nanobody that Targets GFP or mCherry in Budding Yeast

**DOI:** 10.21769/BioProtoc.5019

**Published:** 2024-06-20

**Authors:** Yoshitaka Ogawa, Taisei P. Ueda, Keisuke Obara, Kohei Nishimura, Takumi Kamura

**Affiliations:** Department of Biological Science, Division of Natural Science, Graduate School of Science, Nagoya University, Nagoya, Japan

**Keywords:** Auxin-inducible degron (AID), Super-sensitive AID (ssAID), Affinity linker–based super-sensitive AID (AlissAID), Nanobody (Nb), Budding yeast

## Abstract

The Auxin-inducible degron (AID) system is a genetic tool that induces rapid target protein depletion in an auxin-dependent manner. Recently, two advanced AID systems—the super-sensitive AID and AID 2—were developed using an improved pair of synthetic auxins and mutated TIR1 proteins. In these AID systems, a nanomolar concentration of synthetic auxins is sufficient as a degradation inducer for target proteins. However, despite these advancements, AID systems still require the fusion of an AID tag to the target protein for degradation, potentially affecting its function and stability. To address this limitation, we developed an affinity linker–based super-sensitive AID (AlissAID) system using a single peptide antibody known as a nanobody. In this system, the degradation of GFP- or mCherry-tagged target proteins is induced in a synthetic auxin (5-Ad-IAA)–dependent manner. Here, we introduce a simple method for generating AlissAID strains targeting GFP or mCherry fusion proteins in budding yeasts.

Key features

• AlissAID system enables efficient degradation of the GFP or mCherry fusion proteins in a 5-Ad-IAA–depending manner.

• Transforming the pAlissAID plasmids into strains with GFP- or mCherry- tagged proteins.

## Background

Targeted protein degradation (TPD) is a powerful tool for investigating protein function by rapidly depleting the target proteins in cells. Auxin-inducible degron (AID) is a TPD system that enables auxin-dependent AID-tagged target protein degradation [1]. This technique has been widely used in various eukaryotic species, including yeast, *Drosophila, Caenorhabditis elegans*, and vertebrate cells [2–5]. In this system, expression of the AID-tagged target protein and the auxin receptor Oriza sative TIR1 (OsTIR1) are necessary for target degradation. The OsTIR1 interacts with the AID degron, an interaction that is stabilized by auxin. It further recruits the E3 ubiquitin ligase Skp1-Cul1-F-box (SCF), which leads to the ubiquitination and degradation of target proteins.

The conventional AID system requires a concentration of 100 µM or higher of auxin for the target protein degradation. However, such a high dose of auxin may lead to cytotoxicity [6]. To address this issue, two alternative systems, super-sensitive AID (ssAID) [7] and AID2 [8] have been developed using the bump and hole technique. The ssAID system uses a high-affinity pair of synthetic auxin, 5-Adamantyl-IAA (5-Ad-IAA), and a modified auxin receptor, OsTIR1^F74A^. This advanced system enables the degradation of AID-tagged target proteins at nanomolar concentrations of 5-Ad-IAA.

**Figure 1.**
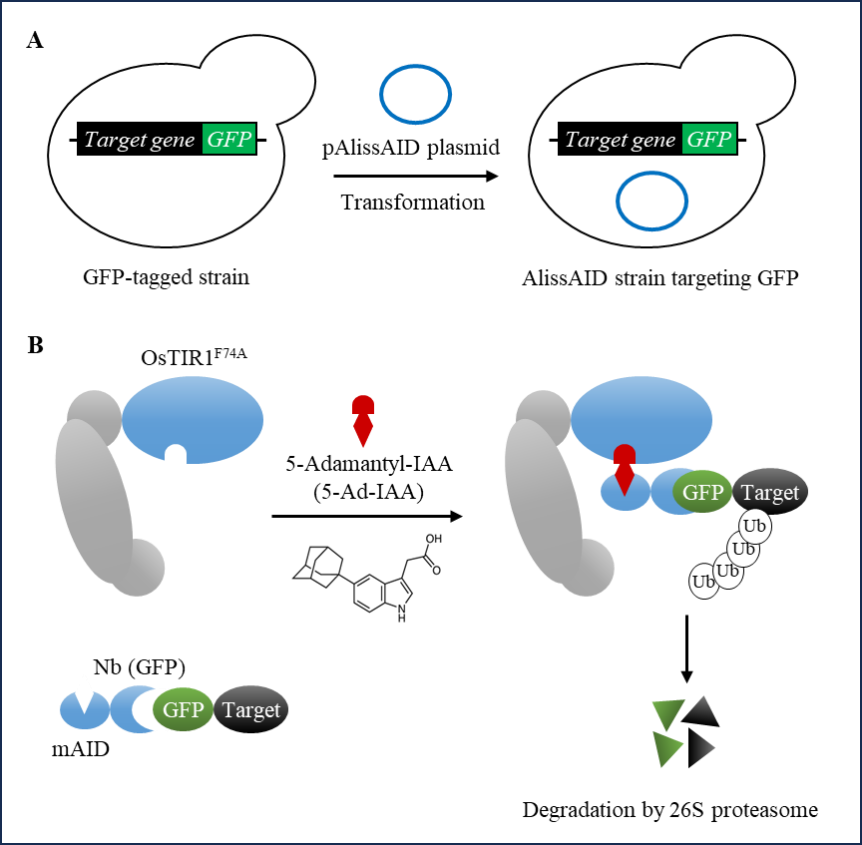
Schematic illustration of generating AlissAID strains from GFP-tagged strains. (A) To generate the AlissAID strain, the pAlissAID anti-GFP plasmid is transformed into a GFP-tagged strain. (B) OsTIR1^F74A^ and mAID-Nb (GFP) are constitutively expressed from the pAlissAID anti-GFP plasmid. The expressed OsTIR1^F74A^ forms the SCF-OsTIR1^F74A^ complex and then polyubiquitinates the GFP fusion protein in a 5-Ad-IAA dependent manner.

Recently, we developed an affinity-linker–based supersensitive AID (AlissAID) system using a single polypeptide antibody known as a nanobody (Figure 1) [9]. In this system, the addition of 5-Ad-IAA induces the degradation of GFP- or mCherry-tagged proteins by stabilizing the interaction between OsTIR1^F74A^ and the minimized AID-tagged nanobody (mAID-Nb). [9]. The binding of OsTIR1^F74A^ and mAID-tag depends on the concentration of 5-Ad-IAA. This advancement enables the use of GFP or mCherry proteins as degradation tags instead of the conventional AID tags in the AlissAID system.

GFP and mCherry are well-known fluorescent tags used in various eukaryotic cells. In budding yeast, GFP fusion with endogenous proteins is easily achievable through genetic manipulation. Furthermore, the GFP tag can be employed in a Yeast GFP Clone collection [10], with a GFP-tagged Open Reading Frame (ORF) at its chromosomal locus, containing 75% of the yeast proteome. Here, we describe a protocol for easy AlissAID strain generation from GFP- or mCherry-tagged strains in budding yeast.

## Materials and reagents

**Biological materials**

1. AlissAID plasmids targeting GFP fusion proteins
*Note: These plasmids are available from National BioResource Project (NBRP)– Yeast*
https://yeast.nig.ac.jp/yeast/top.xhtml
  pAlissAID anti-GFP_313 (*HIS3* marker) (NBRP, catalog number: BYP10393)
  Vector map: https://yeast.nig.ac.jp/yeast/pdf/byp/BYP10393.pdf
  pAlissAID anti-GFP_314 (*TRP1* marker) (NBRP, catalog number: BYP10394)
  Vector map: https://yeast.nig.ac.jp/yeast/pdf/byp/BYP10394.pdf
  pAlissAID anti-GFP_315 (*LEU2* marker) (NBRP, catalog number: BYP10395)
  Vector map: https://yeast.nig.ac.jp/yeast/pdf/byp/BYP10395.pdf
  pAlissAID anti-GFP_316 (*URA3* marker) (NBRP, catalog number: BYP10392)
  Vector map: https://yeast.nig.ac.jp/yeast/pdf/byp/BYP10392.pdf
2. AlissAID plasmids targeting for mCherry fusion proteins
  pAlissAID anti-mCherry_313 (*HIS3* marker) (NBRP, catalog number: BYP10396)
  Vector map: https://yeast.nig.ac.jp/yeast/pdf/byp/BYP10396.pdf
  pAlissAID anti-mCherry_314 (*TRP1* marker) (NBRP, catalog number: BYP10397)
  Vector map: https://yeast.nig.ac.jp/yeast/pdf/byp/BYP10397.pdf
  pAlissAID anti-mCherry_315 (*LEU2* marker) (NBRP, catalog number: BYP10398)
  Vector map: https://yeast.nig.ac.jp/yeast/pdf/byp/BYP10398.pdf
  pAlissAID anti-mCherry_316 (*URA3* marker) (NBRP, catalog number: BYP10399)
  Vector map: https://yeast.nig.ac.jp/yeast/pdf/byp/BYP10399.pdf
3. Budding yeast strains (commonly used strains are useable, BY4741 W303-1a, etc…)
4. Antibodies (anti-OsTIR1 (MBL, catalog number: PD048), anti-Pgk1 (our Laboratory), anti-AIDtag (gifted from Prof. Karim Labib)

**Reagents**

1. Lithium acetate (LioAc) (Wako, catalog number: 127-01545)
2. Salmon sperm DNA (ssDNA) (Wako; catalog number: 043-31381)
3. Poly-ethylene glycol 4,000 (PEG) (Wako, catalog number: 162-09115)
4. Dimethyl sulfoxide (DMSO) (Wako, catalog number: 043-07216)
5. 5-Adamantyl-IAA (5-Ad-IAA) (Tokyo Chemical Industry (catalog number: A3390).
6. D-Glucose (Wako, catalog number: 045-31167)
7. Extract yeast dried (Nacalai Tesque, catalog number: 15838-45)
8. Polypeptone (Wako, catalog number: 398-02117)
9. Adenine hydrochloride (Biosynth, catalog number: FA02944)
10. Yeast nitrogen base without amino acids (ForMedium, catalog number: CYN0410)
11. SC Quadruple Drop Out: -His, -Leu, -Trp, -Ura (ForMedium, catalog number: DSCK1027)
12. Agar (Nacalai Tesque, catalog number: 01028-85)

**Solutions**

1. YPD medium (see Recipes)
2. SD medium (see Recipes)
3. Amino acids and Uracil solution (see Recipes)
4. LiOAc solution (0.1 M, 1 M) (see Recipes)
5. Single-strand DNA (ssDNA; 2.0 mg/mL) (see Recipes)
6. PEG (50% w/v) (see Recipes)
7. 5-Ad-IAA (see Recipes)

**Recipes**

1. YPD medium
  **Table:** 

Reagent	Final concentration	Amount
D-glucose	2%	20 g
Extract yeast dried	1%	10 g
peptone	2%	20 g
Adenine hydrochloride	0.01%	100 mg
MILLI-Q		
Total		1,000 mL
2. **SD medium**
  **Table:** 

Reagent	Final concentration	Amount
D-glucose	2%	20 g
Adenine hydrochloride	0.01%	100 mg
Yeast nitrogen base without amino acids	0.69%	6.9 g
SC Quadruple Drop Out: -His, -Leu, -Trp, -Ura	0.06%	0.6 g
5 M NaOH	5 mM	1 mL
agar (for plate)	2%	20 g
Amino acids and Uracil solution		10 mL
MILLI-Q		
Total		1,000 mL
  For auxotrophic selection, supplement appropriate amino acids and Uracil.
3. **Amino acids and Uracil solution**
  Histidine solution 10 g/L
  Leucine solution 12 g/L
  Tryptophan solution 10 g/L
  Uracil solution 2 g/L
4. **LiOAc solution (0.1 M, 1 M)**
  Dissolve in MILLI-Q at the prescribed concentration and autoclave.
5. **Single-strand DNA (ssDNA; 2.0 mg/mL)**
  Dissolve in MILLI-Q at a concentration of 2.0 mg/mL and autoclave.
6. **PEG (50% w/v)**
  Dissolve in MILLI-Q water at 50% (w/v) and autoclave. The transformation efficiency will decrease if used for a long time; therefore, approximately 10 samples should be prepared at a time.
7. **5-Ad-IAA**
  Dissolve in DMSO at a concentration of 5 mM and store at -30 °C. When it is used for liquid medium, add 1/1,000 of the amount of 5 mM 5-Ad-IAA to the medium. When it is used for plate media, autoclaved media should be cooled to approximately 50 °C before adding 5-Ad-IAA. 5-Ad-IAA containing plates can be stocked at 4°C, hidden from direct light, for at least one month.

**Laboratory supplies**

1. Laboratory disposables:
  - 1.5 mL tubes (Watson, catalog number: 131-7155C)
  - 15 mL tubes (Greiner, catalog number: 188 271- 013)
  - 50 mL tubes (Greiner, catalog number: 227 261)
  - 1000 μL tips (Watson, catalog number: 110-706C)
  - 200 μL tips (Watson, catalog number: 110-705C)
  - Petri dishes (STAR, catalog number: RSU-SD9015-2)

## Equipment

1. Cool incubator (As one, catalog number: A1201)
2. Shaking incubator (N-BIOTEK, catalog number: NB-205L)
3. Heat block (WAKENYAKU, catalog number: WKN-9626)
4. Vortex (Scientific Industries, Inc., catalog number: SI-0286)
5. Centrifuge (Eppendorf, catalog number: 5420000237
6. Microscope (AxioObserver Z1 (Carl Zeiss, Oberkochen, Germany) equipped with a CCD camera (AxioCam MRm; Carl Zeiss))

## Procedure

- **Preparation of GFP- or mCherry-tagged strains**
  Prepare strains in which target proteins are tagged with GFP or mCherry. It is easy to add fluorescent tags to an ORF in its chromosomal location through homologous recombination in budding yeast [10]. Alternatively, the appropriate strains were selected from the GFP Clone Collection [10]. In this paper, we used W303-1a strains that were tagged at the C-terminus of *ASK1* on the genome and selected with *HIS3* using the method previously reported [9].
*Note: Although we have not tested the protein degradation of N-terminal–tagged proteins, in principle, the AlissAID system would work on N-terminal tagged proteins.*
- **Transformation of the pAlissAID plasmid**
*Note: Pay attention to the selection marker; in the case of the GFP Clone Collection, the GFP sequence is inserted at the C-terminus of the target gene using HIS3 as the selection marker. Therefore, we used the pAlissAID plasmids encoding TRP1, LEU2, and URA3 (Figure 2). All these pAlissAID plasmids (HIS3, TRP1, LEU2, and URA3 encoding) are available from NBRP.*
**Day 1**
  1. Grow yeast cells overnight in 5 mL of YPD medium at 30 °C with shaking at 230 rpm.
**Day 2**
  1. Dilute the cells to OD_600_ = 0.3 and regrow the cells in 5 mL of YPD medium at 30 °C with shaking at 230 rpm for 3–5 h.
  2. When cells have grown to approximately OD_600_ = 1.0, centrifuge them (1,000× *g*, 5 min, approximately 25 °C) and discard the supernatant.
  3. Suspend cells in 1 mL of sterilized water and transfer to a sterilized 1.5 mL tube.
  4. Centrifuge (20,000× *g*, 30 s, 25 °C) and discard the supernatant.
  5. Resuspend in 1 mL of 0.1 M LiOAc.
  6. After centrifugation (20,000× *g*, 30 s, 25 °C), discard the supernatant and centrifuge again to completely remove the supernatant.
  7. Denature the ssDNA at 90 °C for approximately 5 min and cool on ice.
  8. Add the following solutions and mix using a vortex.
    2.0 mg/mL ssDNA, 25 μL
    pAlissAID plasmid, 1 μg
    sterilized water, up to 50 μL
  9. Add 240 μL of 50% w/v PEG and 36 μL of 1 M LiOAc. Vortex the mixture and incubate in a heat block at 30 °C for 30 min.
  10. Add 40 μL of DMSO. Vortex the mixture and incubate in a heat block at 42 °C for 20 min.
  11. Centrifuge (20,000× *g*, 30 sec, room temperature) and discard the supernatant.
  12. Resuspend the pellet in the remaining supernatant (at least 50 μL is needed) and plate the cells onto an SD plate medium (lacking histidine, leucine, tryptophan, or uracil).
  13. Incubate at 30 °C for 2–3 days.
    **Figure 2.**
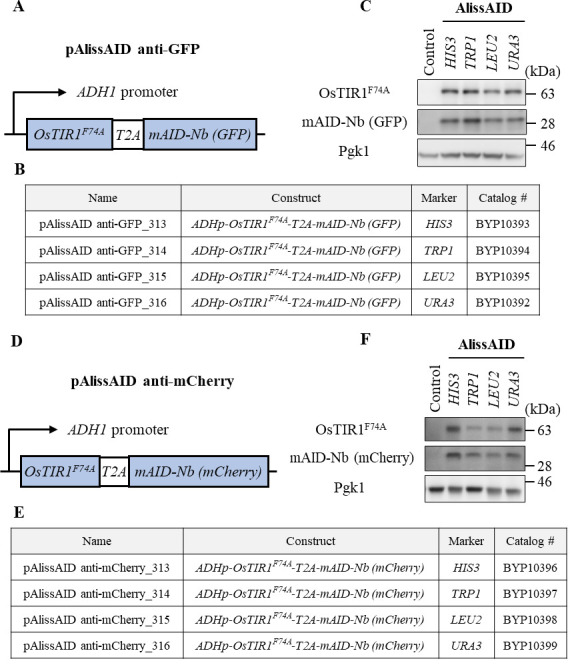
Overview of pAlissAID plasmids. (A, D) Schematic illustrations of pAlissAID (anti-GFP or anti-mCherry) plasmids. pAlissAID plasmids contain an expression cassette of OsTIR1^F74A^-T2A-mAID-Nb (GFP or mCherry) under the control of constitutive *ADH1* promoter. The expressed protein is cleaved at the self-cleavage site of T2A to produce OsTIR1^F74A^ and mAID-Nb, separately. (B, E) Lists of pAlissAID plasmids. Each plasmid has a different selection marker *HIS3, TRP1, LEU2*, or *URA3*. (C, F) Immunoblot analysis of OsTIR1^F74A^ and mAID-Nb. Each pAlissAID plasmid with a different selection marker was transformed into the W303-1a cells to express both OsTIR1^F74A^ and mAID-Nb. Pgk1 was used as a loading control.
**Day 4 or 5**
  1. Pick up the colonies that contain pAlissAID plasmids (Figures 3A and 4A).
    **Figure 3.**
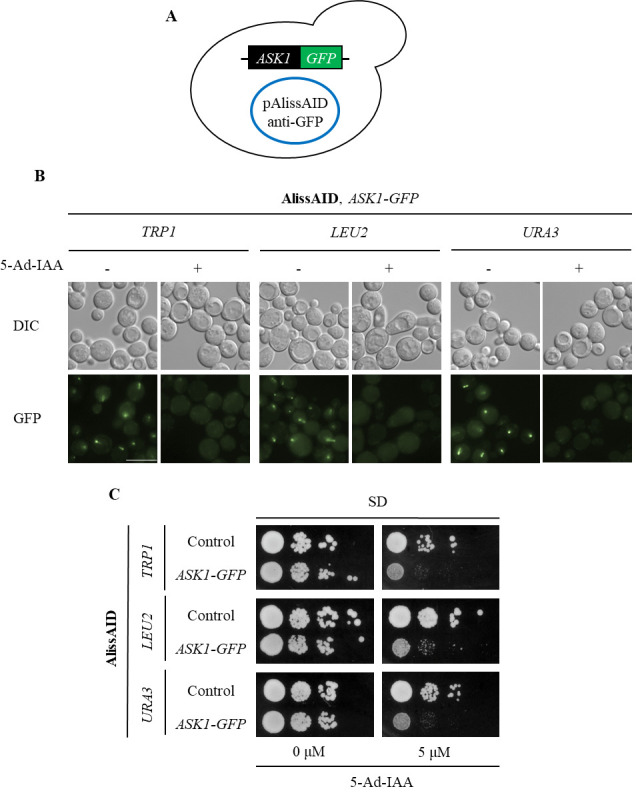
Degradation of GFP fusion proteins in AlissAID strains. (A) AlissAID strains were generated by the transformation of pAlissAID plasmids with a different selection marker (*TRP1, LEU2*, or *URA3*) into the Ask1-GFP strain. (B) Microscopic observations of fluorescent signals of Ask1-GFP in AlissAID strains. Cells were treated with or without 5.0 µM 5-Ad-IAA for 3 h at 30 . Scale bar, 10 µm. (C) Serial dilution spotting of control and AlissAID strains on SD medium plate with or without 5.0 µM 5-Ad-IAA. 10-fold serial dilutions were made from the cells with OD_600_ = 0.3 and spotted into the plate. The strain that expresses Ask1 instead of Ask1-GFP and contains pAlissAID plasmid is used as a control.
    Cells were grown for 48 h at 30 .
    **Figure 4.**
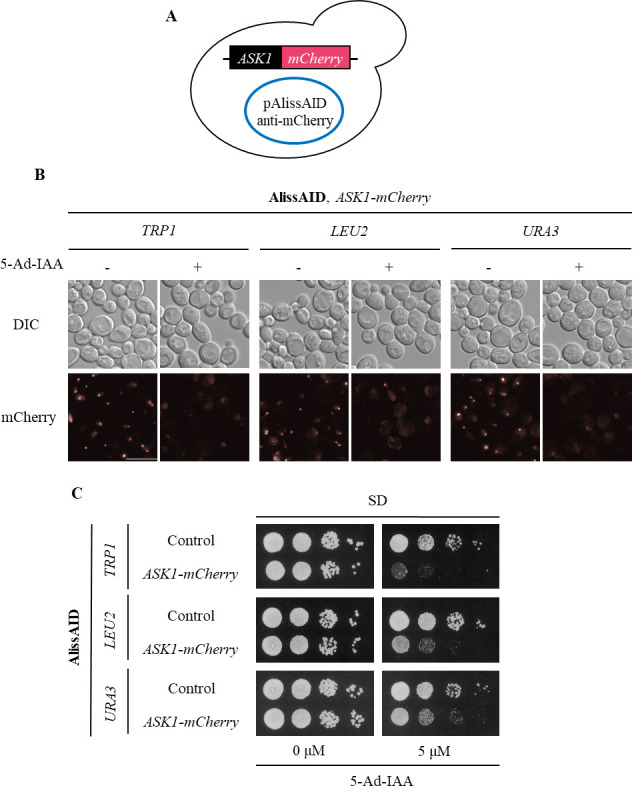
Degradation of mCherry fusion proteins in AlissAID strains. (A) AlissAID strains were generated by the transformation of pAlissAID plasmids with a different selection marker (*TRP1, LEU2*, or *URA3*) into the Ask1-mCherry strain. (B) Microscopic observations of fluorescent Ask1-mCherry signals in AlissAID strains. Cells were treated with or without 5.0 µM of 5-Ad-IAA for 3 h at 30 . Scale bar, 10 µm. (C) Serial dilution spotting of control and AlissAID strains on SD medium plate with or without 5.0 µM 5-Ad-IAA. 10-fold serial dilutions were made from the cells with OD_600_ = 0.3 and spotted into the plate. The strain that expresses Ask1 instead of Ask1-mCherry and contains pAlissAID plasmid is used as a control. Cells were grown for 48 h at 30 .
  2. After picking up the colonies and growing them in SD medium, confirm successful plasmid transformation into the yeast by any method (Immunoblot, microscope observation, phenotyping, etc.) (Figure 3B and C, 4B and C).
*Note: Expressed nanobodies bind to GFP or mCherry instead of target protein in the AlissAID strains. It is unlikely that these nanobody interfere with the target protein’s function.*
**5-Ad-IAA treatment**
  When it is used for liquid medium, add 1/1,000 of the amount of 5 mM 5-Ad-IAA to the medium (final concentration 5 μM of 5-Ad-IAA). When it is used for plate media, autoclaved media should be cooled to approximately 50 °C before adding 5-Ad-IAA.

**Check the protein degradation by fluorescence microscopy**

Degradation of target proteins can be confirmed by immunoblot or fluorescence microscopy. If the target is an essential protein, it can also be verified by a serial dilution assay in 5-Ad-IAA–containing plate medium. These detailed methods are described in the original paper [9]. Here, we briefly introduce an example of verification using fluorescence microscopy.

**Day 1**

1. Grow yeast cells overnight in 5 mL of SD medium at 30 °C with shaking at 230 rpm.

**Day 2**

1. Dilute the cells to OD_600_ = 0.3 and regrow the cells in 5 ml of SD medium at 30 °C with shaking at 230 rpm for 3–5 h.
2. When the cells have grown to approximately OD_600 _=1.0, add 5 μL of 5 mM 5-Ad-IAA and incubate at 30 shaking at 230 rpm for 1–3 h.
3. Collect the cells and observe fluorescent signals of live cells by using a fluorescence microscope.
*Note: PFA fixation by using 4% paraformaldehyde is also available for microscopic observation.*
*Note: The efficiency of protein degradation depends on the expression level and localization of the target protein, so in some cases immunoblots would be more suitable for confirming protein reduction.*

## Data analysis

**Representative data**

AlissAID strains induce target protein depletion in the presence of 5 µM of 5-Ad-IAA (Figures 3B and 4B). When targeting essential genes, the AlissAID strains exhibited severe growth defects in plates containing 5-Ad-IAA (Figures 3C and 4C). Immunoblot, microscopic observation, and serial dilution assay methods followed those detailed in the original paper [9].

## Validation of protocol

This protocol or parts of it has been used and validated in the following research article:

Ogawa et al. (2023). Development of AlissAID system targeting GFP or mCherry fusion protein. PLOS Genetics (Figure 5).
